# Indoor flow datasets of two-layered cross-ventilation models by particle image velocimetry and hot wire anemometry

**DOI:** 10.1016/j.dib.2023.109856

**Published:** 2023-11-26

**Authors:** W. Wang, N. Ikegaya, C. Hirose, M.F. Mohamad

**Affiliations:** a Faculty of Engineering Sciences, Kyushu University, Japan; b School of Mechanical Engineering, College of Engineering, Universiti Teknologi MARA, Malaysia

**Keywords:** Two velocity components, Approaching flow, Incidental flow, PIV, HWA, Wind-tunnel experiment, CFD validation

## Abstract

This data article provides temporally and spatially high-resolution datasets of the indoor velocity fields for cross-ventilation models of two-layered simplified buildings separated by a second floor at the middle height with an opening using wind-tunnel experiments. The datasets are based on the research article entitled “Quantifying natural cross-ventilation flow of a two-layered model used for terraced houses in tropical zones by particle image velocimetry” by Ali et al. [1]. Two cases are considered based on the positions of the inlet and outlet openings on each floor. The measurements were conducted using hot-wire anemometry (HWA) with 10,000 Hz and particle image velocimetry (PIV) with 1000 Hz for a sufficiently long period to determine reliable statistics of the mean, variances, and covariances. In addition, the article provides the instantaneous datasets of two velocity components determined by PIV for the cross-ventilation models. The datasets can be used for both computational fluid dynamics (CFD) validation and further investigation of turbulent flow nature of the multi-layer cross ventilation flow.

Specifications Table

**Table:** 

Subject	Engineering, Environmental Engineering
Specific subject area	Cross Ventilation, Wind Induced Ventilation, Indoor Air Quality
Data format	Raw, Analyzed
Type of data	Table (Microsoft ^Ⓡ^ Excel for Mac. Version 16.78, Binary format), Graph (Microsoft ^Ⓡ^ Excel for Mac. Version 16.78, Binary format), Binary (netCDF created by netCDF library version 4.3.3)
Data collection	For hot-wire anemometry (HWA), an x-type hot-wire probe (0249R-T5, KANOMAX JAPAN, INC.), constant temperature amplifier unit (MODEL 1011, KANOMAX JAPAN, INC) were employed. For particle image velocimetry (PIV), a high-speed camera (Phantom T1340, Nobby Tech. Ltd.), double-pulse laser device (Tolar-527–20, Beamtech Optronics Co, Ltd.), seeding device (CTS-1000, SEIKA Digital Image Corporation) were employed. The particle images were analyzed by a PIV postprocessing software, Koncerto-II Version K2_2.2.37.33 (64bit) (SEIKA Digital Image Corporation).
Data source location	Institution: Tokyo Polytechnic University City/Town/Region: Atsugi, Kanagawa Country: Japan
Data accessibility	Repository name: Mendeley Data Data identification number: DOI:10.17632/bmf5kf273b.1 Direct URL to data: https://data.mendeley.com/datasets/bmf5kf273b/1
Related research article	A.M. Ali, M.F. Mohamad, W. Wang, C. Hirose, R. Yoshie, N. Ikegaya (2023) Quantifying natural cross-ventilation flow of a two-layered terraced house used for terraced houses in in tropical zones by particle image velocimetry, Building and Environment 244, 110829, https://doi.org/10.1016/j.buildenv.2023.110829

## 1 Value of the Data

• The temporally and spatially high-resolution datasets can be used by researchers who propose evaluation indices of indoor air quality for cross-ventilation flow of a multi-layered simplified building [1].
• The velocity datasets can be used by researchers, engineers, and CFD users in the field of indoor environmental study to validate the simulations for a simplified multi-layer indoor ventilation flow.
• The datasets can be used by CFD users as a benchmark case for cross-ventilation flow of a multi-layer simplified building to develop CFD techniques such as numerical settings and turbulence models [2,3].
• The instantaneous velocity datasets can be utilized by researchers and CFD-software developers for developing a new validation process of unsteady CFDs by determining probability density, power spectrum, and low-occurrence velocity events [4], [5], [6], [7], [8].
• The datasets providing two different measurement techniques can be used by experimenters to justify their experiments for cross-ventilation flow.
• The statistical and instantaneous velocity datasets can be employed by data scientists as an input and output data of inductive approaches such as regression analysis and machine learning method to predict indoor velocity distributions.

## 2 Data Description

There are three Excel files and eight binary files following netCDF format. For the definition of the variable and coordinate system, please refer to Section 2. The model case names, U2D1 and U1D2 are explained in Section 2.2.

(1) Approaching and incidental wind profiles (prof01-inflow.xlsx)

The Excel file is the list of the temporally averaged streamwise velocity component, variances and covariance of the streamwise and vertical velocity component. The Excel file has two sheets for the data at the approaching flow position (“ApproachingFlow”) and incidental flow position (“IncidentalFlow“). See Fig. 3 for the definition of the positions. The graphs of the data are also included in each sheet. The coordinates and velocity are normalized by H and $u_{H}$, respectively, where H=200mm is the model height and $u_{H}$ is the mean streamwise velocity component at the building height of the incidental flow.

(2) Velocity component profiles for the model U2D1 (prof02-U2D1.xlsx)

The Excel file is the list of the temporally averaged streamwise velocity component, variances of the streamwise and vertical velocity components at several measurement locations. The definition of the measurement locations is explained in Fig. 3. The file has two sheets for the HWA (“HWA”) and PIV (“PIV”). The graphs of the data are also included in each sheet.

(3) Velocity component profiles for the model U1D2 (prof03-U1D2.xlsx)

The same format data as prof02-U2D1.xlsx but for the model U1D2.

(4) Two-dimensional datasets of the velocity component statistics by PIV (U2D1.stat.nc)

The binary file follows the netCDF format. It includes the temporally averaged streamwise velocity component, wind speed in a vertical plan, variances and covariances of the streamwise and spanwise velocity components for the model U2D1 obtained by PIV.

(5) Two-dimensional datasets of the velocity component statistics by PIV (U1D2.stat.nc)

The same format data as U2D1.stat.nc but for the model U2D1.

(6) Two-dimensional instantaneous datasets of the two velocity components (U1D2-T1.nc, U1D2-T2.nc, U1D2-T3.nc)

The binary file follows the netCDF format. It includes the instantaneous streamwise and vertical velocity components for the model U1D2 by PIV. The three files with T1, T2 and T3 indicate three different trials. The time in the datasets is normalized by $H/u_{H}$.

(7) Two dimensional instantaneous datasets of the two velocity components (U2D1-T1.nc, U2D1-T2.nc, U2D1-T3.nc)

The same format data as U1D2-T1.nc, U1D2-T2.nc, U1D2-T3.nc but for the model U2D1.

## 3 Experimental Design, Materials and Methods

(1) Experimental design

**Fig. 1:**
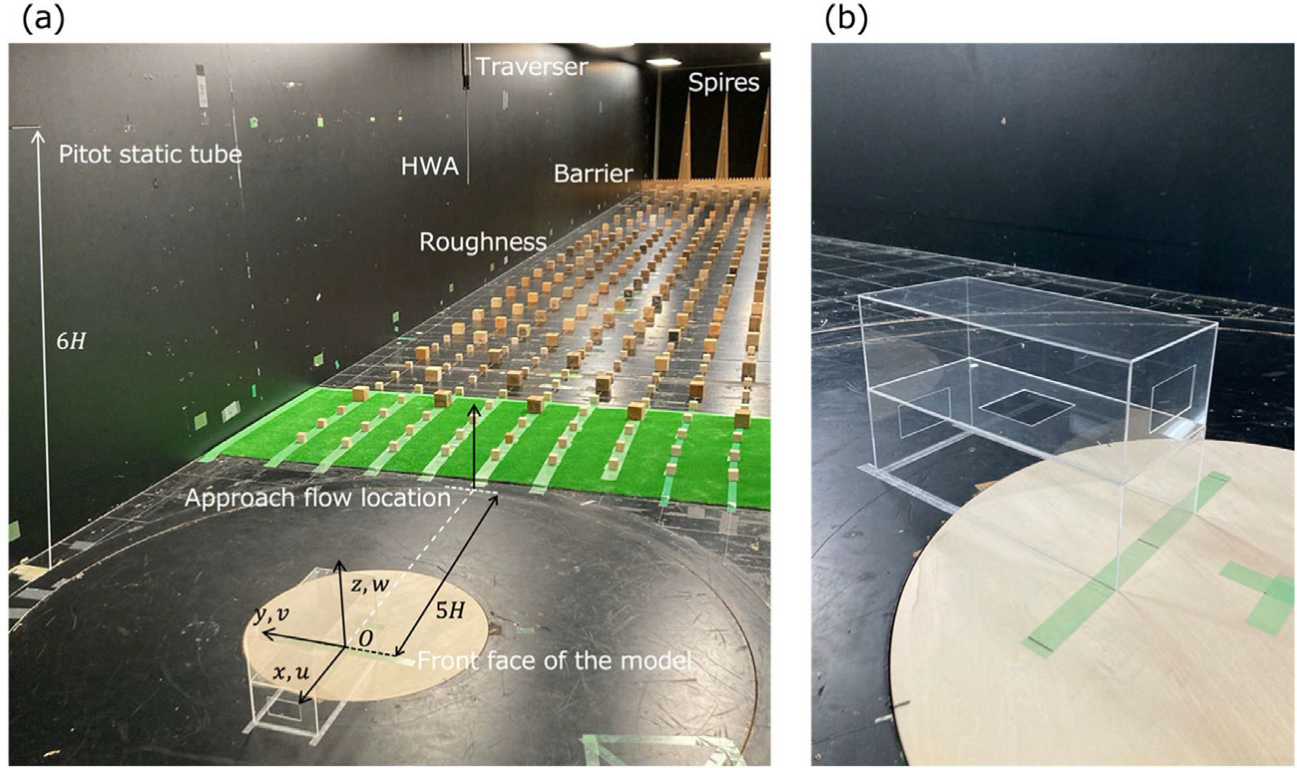
(a) Experimental settings of the turbulence generators (spires, barrier and roughness) and ventilation model and (b) ventilation model (modified from Ali et al. [1]).

An open-circuit wind tunnel at Tokyo Polytechnic University (TPU), Japan, was employed for the experiments. The dimension of the wind tunnel is 19 m in the streamwise, 2.2 m in the spanwise, and 1.8 m in the vertical directions. The definition of the coordinates and velocity components are shown in Fig. 1(a). The approaching flow was measured at 5H upstream position from the origin at the spanwise center. A pitot static tube as shown in Fig. 1(a) was employed to measure the reference wind speed at z/H=6. The reference wind speed was used to derive the streamwise velocity component at the block height, $u_{H}$, based on the vertical profile of the incidental flow. Throughout the datasets,$\;u_{H}$ is used as a scaling velocity. The variation of $u_{H}$ among experiments are less than 0.4 %. The Reynolds number defined by H and $u_{H}$ is approximately $8.6\;\times \;10^{4}$. The approaching flow was generated to follow the power law for the streamwise velocity component with the power index of 0.25 and the exponential equations for the turbulent kinetic energy using roughness, spires, and a barrier in the upstream region of the test section (Fig. 1(a)).

(2) Ventilation model design

**Fig. 2:**
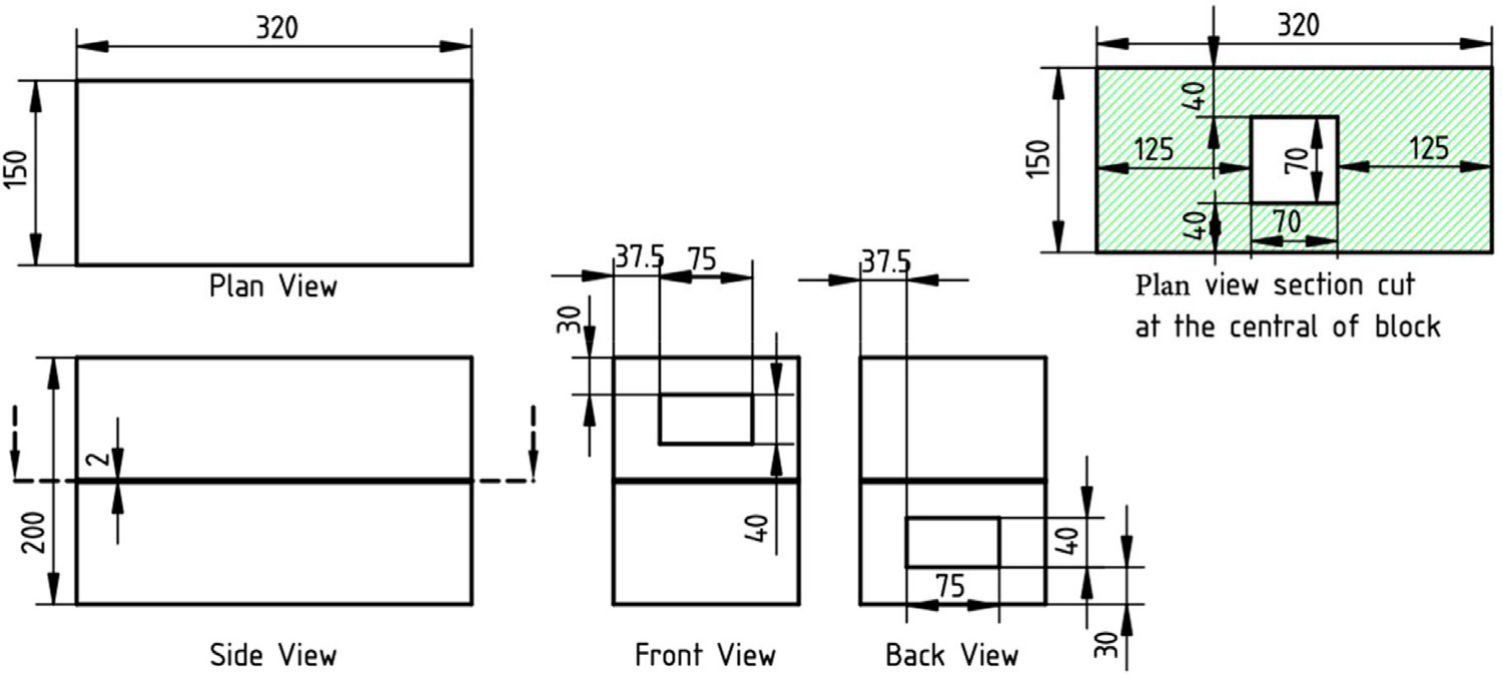
Detailed dimensions of the ventilation model (unit in mm). The front and back views indicate the windward and leeward faces of U2D1, and vice versa for U1D2.

Fig. 1(b) shows the isometric view of the ventilation model. The detailed dimensions of each face are illustrated in Fig. 2. The external dimensions of the model height, length, and width are 200 mm, 320 mm, and 150 mm. The wall thickness is 2mm. There is a divider with an opening of 70 mm × 70 mm at the streamwise and spanwise centers at the middle height of the model. The inlet and outlet openings are the dimensions of 75 mm × 40 mm. Using the same ventilation models, the datasets provide two conditions: the inlet and outlet openings were positioned at the second and first layers, and vice versa (U2D1 and U1D2, respectively).

(3) Data acquisition

**Fig. 3:**
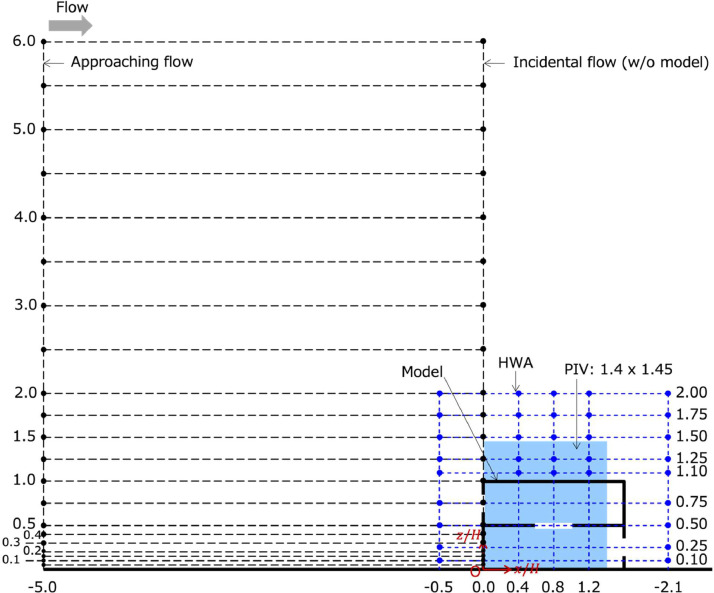
Measurement locations. The filled circles indicate the measurement locations of HWA. The blue shaded area indicates the regions measured by PIV. The model U2D1 is shown in the schematics, but the measurement positions are identical for U1D2. The horizontal and vertical axes were normalized by H.

The vertical profiles of the approaching flow (at x/H=−5 and y/H=0), incidental flow (at x/H=0 and y/H=0 without the model), and five locations near the model were obtained using an x-type HWA (0249R-T5, KANOMAX JAPAN, INC.). A probe calibrator (MODEL 1065, KANOMAX) was used to determine the wire angle in advance for each probe. The fixed wire angles were used for each probe, while the calibration coefficients of the HWA probe were determined each day of the experiments. Fig. 3 shows the measurement positions of the approaching flow, incidental flow, and vertical profiles near the model. The filled circles in Fig. 3 represents the measurement locations. The approaching and incidental profiles were measured at 21 vertical points between z/H=0.1 and 6.0. The velocity profiles around the model were determined at five streamwise positions between z/H=0.1 and 2.0. The measurement period and sampling frequency are 180 s and 10,000 Hz (time interval of 0.0001 s), respectively.

The shaded areas in Fig. 3 shows the region captured by PIV (1.4H in streamwise, and 1.45H vertically). The two velocity components in the xz-plane (namely, u and w) were determined at the spanwise center, y/H=0. A double pulse laser device (Tolar-527-20, Beamtech Optronics Co, Ltd.) was installed at x/H=3.5 (approximately, 3.2 m downstream from the downmost face of the model). The interval of double-pulse laser was 200 μs, and the frame rate was set as 2000 fps (time interval of 0.0005 s). A high-speed camera (Phantom T1340, Nobby Tech. Ltd.) was installed at approximately 1 m from the model on the side. The measurement area in a xz plane was captured with a resolution of 1024 × 1024 pixel. The measurement period of each trial is 11 s and was repeated three times. Therefore, the total sampling duration is 33 s. The expected variations in the mean and standard deviation over 30 s were 1 % and 2 %, respectively (Ikegaya et al. [9])

After obtaining raw images, the velocity components in a xz plane were determined by image preprocessing using a PIV software, Koncerto-II Version K2_2.2.37.33 (64 bit) (SEIKA Digital Image Corporation). After subtracting the minimum brightness distributions from the original images, a three-time multi-pass correlation method with a least-squares Gauss-fit sub-pixel analysis was employed to determine the velocity distribution. The output grid resolution was 16 pixels corresponding to a resolution of approximately 6 mm (The interrogation window size was 32 × 32 pixel with a 50 % overlap.).

(4) Data calculation

The time series data with the sampling period T were used to calculate the mean, variances, and covariance.

(1) $$\bar{u}=\frac{1}{T}\int_{T}u\left(t\right)dt$$

(2) $$\sigma_{u}^{2}=\frac{1}{T}\int_{T}{\left(u\left(t\right)-\bar{u}\right)}^{2}dt$$

(3) $$\sigma_{w}^{2}=\frac{1}{T}\int_{T}{\left(w\left(t\right)-\bar{w}\right)}^{2}dt$$

(4) $$\bar{u^{\prime }w^{\prime }}=\frac{1}{T}\int_{T}\left(u\left(t\right)-\bar{u}\right)\left(w\left(t\right)-\bar{w}\right)dt$$

When measurements were repeated N times, the ensemble average of each statistics $\phi_{i}$ was calculated as follows.

(5) $$\langle \phi \rangle =\frac{1}{N}\Sigma_{i=1}^{N}\phi_{i}$$

Therefore, the statistical datasets provided by HWA is $\bar{u}$, $\sigma_{u}^{2}$, $\sigma_{w}^{2}$, $\bar{u^{\prime }w^{\prime }}$, and those by PIV are $\langle \bar{u}\rangle$, $\langle \sigma_{u}^{2}\rangle$, $\langle \sigma_{w}^{2}\rangle$, $\langle \bar{u^{\prime }w^{\prime }}\rangle$, respectively. All the velocity data, coordinates, and time in the datasets are normalized using H and $u_{H}$.

(5) Accuracy and repeatability

The accuracy of the HAW was quantified by using the probe calibrator. Within the range of the calibration wind speeds between 3.8 m/s to 11.3 m/s, the bias errors of the HWA were 1.5 % for u component and 2.9 % for w component. For both HWA and PIV were repeated three times to confirm the repeatability of the statistics. As for HWA, the variation in three trials (for approaching flow measurements) were follows: $\bar{u}/u_{H}≅0.5$%, $\sigma_{u}^{2}/u_{H}^{2}≅\sigma_{w}^{2}/U_{H}^{2}≅0.01$%, and $\bar{u^{\prime }w^{\prime }}/u_{H}^{2}≅4.8$%, respectively. Three trial variations of PIV were $\bar{u}/u_{H}≅2$%, and $\sigma_{u}^{2}/u_{H}^{2}≅\sigma_{w}^{2}/u_{H}^{2}≅0.2$%.

## Limitations

Both datasets of PIV and HWA do not include the spanwise velocity components. The indoor velocity components were measured only by PIV. The measurement period of 33 s for PIV is shorter than that of HWA, while HWA datasets provide 180-s data at each point.

## Ethics Statement

The current work does not involve human subjects, animal experiments, or any data collected from social media platforms.

## CRediT authorship contribution statement

**W. Wang:** Software, Investigation, Formal analysis. **N. Ikegaya:** Conceptualization, Writing – original draft, Writing – review & editing, Funding acquisition, Supervision. **C. Hirose:** Conceptualization, Investigation, Funding acquisition. **M.F. Mohamad:** Conceptualization, Funding acquisition, Supervision.

## Data Availability

Velocity datasets of a simplified two-layered terraced house by WTE (Original data) (Mendeley Data)
